# Numerical study of ice loads on different interfaces based on cohesive element formulation

**DOI:** 10.1038/s41598-023-41618-z

**Published:** 2023-09-02

**Authors:** Wenqiang Xing, Shengyi Cong, Xianzhang Ling, Xinyu Li, Zhihe Cheng, Liang Tang

**Affiliations:** 1 School of Civil Engineering, Harbin Institute of Technology, Harbin, 150090 Heilongjiang China; 2 State Key Laboratory of Frozen Soil Engineering, Cold and Arid Regions Environmental and Engineering Research Institute, Chinese Academy of Sciences, Lanzhou, 730000 Gansu China

**Keywords:** Civil engineering, Mechanical engineering

## Abstract

With the increase of marine activities in the Arctic area, the demand for reliable design of marine structures is growing. Numerous publications can be found regarding simulations of ice action on structures using cohesive element models of the ice. However, previous studies have rarely discussed the influence of structural form, that is, the form of ice-structure interaction interface, on the ice load. Thus, a more comprehensive understanding of the ice load on structures with different interface geometries needs to be explored. In the present paper, three-dimensional finite element models with the cohesive element method are developed to investigate the ice load on different structures. The numerical results are validated based on in-situ testing data and the results of the previous numerical model. Parametric studies considering structure widths, inclination angles, ice velocity as well as structure roughness are conducted to explore the horizontal force and failure process of the ice sheet. The process of ice-structure interaction and ice loads on different structural forms were discussed and simplified diagrams of ice load distribution on the interface were developed.

## Introduction

Sea ice in the Arctic and Subarctic regions poses a great risk to the safety of marine structures^1^. The growth of human activities in the Arctic and Subarctic regions leads to an increase of demands and challenges in engineering design of structures against ice-induced forces. Although researches related to ice-structure interactions have been carried out many years ago, engineering accidents induced by ice load still occur from time to time. Investigation on ice-structure interactions is hence an important prerequisite for the safety of human activities in these extreme cold regions.

Prediction of the ice load on man-made structures, such as vessels, offshore structures, lighthouses, etc., is of particular concern for the structural design and Maintenance^2^. Many studies have been conducted to obtain the ice load on column structures and conical structures subjected to level ice^3–8^. ISO199006 shows the design method of ice load based on vertical structure and inclined structure. In the existing studies, the single structure form has received more attention, but the comparison of different structure shapes, especially the discussion of the difference of the distribution of ice load on the structure with finite size needs further investigation. When ice interacts with conical structures, different ice-structure interfaces can be formed. For instance, a sloping curved surface or a level sharp corner can be formed under different water levels. However, engineering structures have different geometric forms due to their functions or safety requirements, which results in diverse interfaces in ice-structure interactions. A more comprehensive understanding of the ice load on structures with different ice interfaces is required and should be explored.

The in-situ and scaled model tests provide a large amount of ice load data in actual environmental conditions, which helps in understanding the progress of ice-structure interactions and failure of ice^9–19^. However, in-situ and model tests are time-consuming and expensive, and there are high requirements on test equipment and site conditions^20^. Development of simulation methods, e.g., finite element method (FEM), discrete element method (DEM) and lattice method, has been an interesting alternative that could save both time and cost, and offer a great number of test parameters under controlled conditions^21^. To effectively capture the fracture behavior, fracture mechanics was introduced in the ice-structure interaction^22,23^. Mulmule and Dempsey^24^ applied cohesive element to ice mechanics analysis to provide a new approach for sea ice fragmentation problems. The cohesive element method (CEM) relies on the mathematical framework of conventional FEM and can explicitly describe the viscous cracks and fracture process of ice^25^. As a convenient and reliable numerical method, the CEM has been widely used in fracture simulation of ice-structure interaction^26–30^.

In this paper, a three-dimensional (3D) finite element model with CEM was developed based on ABAQUS/EXPLICIT to investigate the ice load on various structures. The numerical model was validated with the in-site measured data of a load event on Norströmsgrund lighthouse. Three basic interfaces including cylindrical surface (CS), plate surface (PS) and corner interface (CI) were considered to investigate the ice load on structures with different widths and inclination angles. And simulation cases were performed to evaluate the influence of ice velocity and structure roughness. The process of ice-structure interaction and ice loads on different structural forms were explored. Simplified diagrams of ice load distribution on the interface were developed which would be a reference for engineering structure design.

## Cohesive element method and constitutive relationships

**Figure 1:**
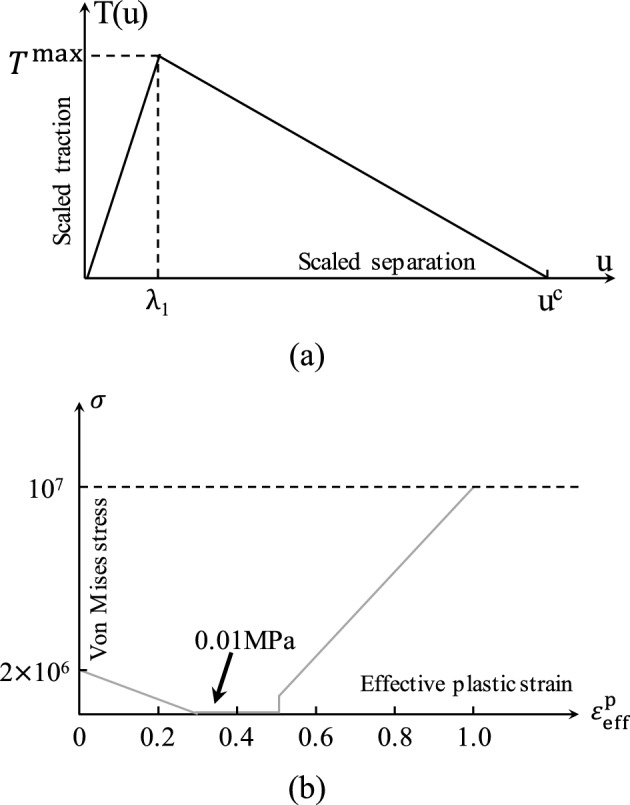
(**a**) The Traction Separation Law (TSL) curves applied in cohesive element method (CEM); (**b**) Hardening curve of elastoplastic constitutive model^33,34^.

In this study, the ice sheet is modeled using the CEM. The interaction region of the ice sheet is discretized with ice bulk elements which are connected by cohesive elements. The traction separation law (TSL) curve used in CEM depicts the relationship between the crack separation *u* of cohesive elements and the traction stress *T*^26^. A linear softening criterion (also known as bilinear criterion) is shown in Fig. 1a. At the beginning of cracking, the cohesion element is usually considered to be linear. When the traction stress reaches the failure stress (represented by *T*^max^ in Fig. 1a), the microcrack is initiated and the material begins to soften from this moment. The damage development stage of the cohesive element describes the process of material stiffness decreasing with the development of crack. It can be seen that the stress decreases linearly with the amount of separation at the crack interface. When the separation reaches the maximum interface separation *u*^c^, the cohesive element is deleted, and the crack is formed.

The ice bulk element is usually assumed to be an elastic–plastic material, and the plastic deformation reflects the microstructure changes of ice, such as recrystallization and pressure melting. The Mohr–Coulomb is suggested in ISO19906^31^ to estimate the strength of ice rubble. However, it has been reported that MC model is not suitable for estimating the shear strength of ice at high internal friction angles^32^. In this study, an isotropic elastic–plastic material model (Fig. 1b) proposed by Hilding et al.^33,34^ was employed to simulate the behavior of the ice bulk element. The material is set to harden to 50% plastic strain (Fig. 1b) in order to prevent numerical instabilities due to distorted elements. Heavily distorted element with plastic strain exceeding 0.7 are removed to save CPU time. Material parameters are defined in Table 1^28^.

**Table 1:** Material parameters for ice elements and cohesive elements^28^.

	Description	Value
Ice bulk element	Elastic modulus (GPa)	5
	Poisson’s ratio	0.3
	Density (kg/m^3^)	900
Cohesive element	Elastic stiffness (normal/shear1/shear2) (GPa)	5/5/5
	Initial damage stress (normal/shear1/shear2) (MPa)	1.5/1.1/1.1
	Energy release rate (normal/shear1/shear2) (N/m)	100/100/100
	Density (kg/m^3^)	900

## Numerical modeling of ice sheet interacting with the Norströmsgrund lighthouse

### Norströmsgrund lighthouse model

**Figure 2:**
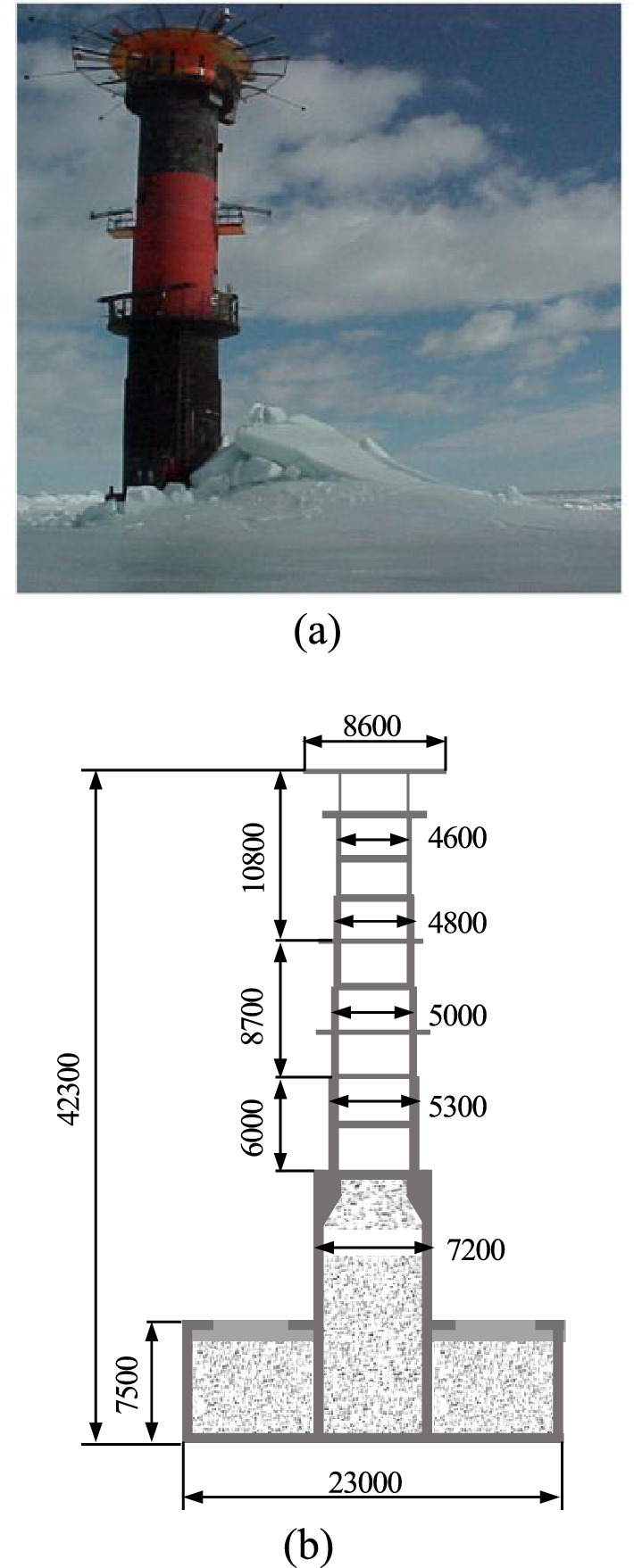
(**a**) The Norströmsgrund lighthouse^35^; (**b**) Profile of lighthouse^36^.

**Figure 3:**
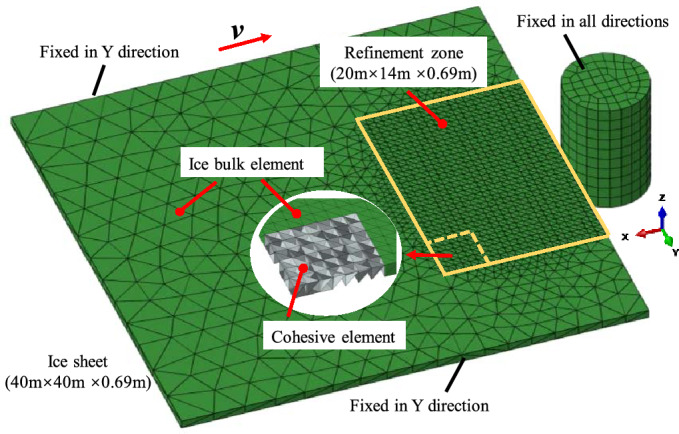
Scenario of collision between ice sheet and the lighthouse.

A full-scale simulation of the interaction between the Norströmsgrund lighthouse^35^^,^^36^ (Fig. 2) and a flat ice block is performed using the ABAQUS finite element code. The CEM is used for 3D modeling of ice sheet, as shown in Fig. 3.

In order to verify the model reliability, the basic working conditions of the model are taken from simulation parameters reported by Hilding et al^33^. According to the actual monitoring data, the overall dimension of the ice model is 40 m × 40 m and its thickness is 0.69 m. In order to reduce computational costs and based on the scale of interaction between structure and sea ice, the cohesive unit area is set to be 20 m × 14 m. The model grid affects breakage morphology of the sea ice and load amount. The hexahedral grid produces a zigzag crack path, whose size is $\sqrt{2}$ times larger than the actual crack size and results in additional energy loss^6^. It is difficult to eliminate this effect by mesh refinement. Accordingly, the ice model mesh is generated by tetrahedral elements (C3D4), and the cohesive element (COH3D6) is inserted into the ice^28^. The section at sea level is selected for the lighthouse structure. Because of large overall stiffness difference of the structure, the lighthouse structure is considered as a rigid body.

### Buoyancy model and collision scenario

The normal displacement is constrained by smooth lateral boundaries of the ice, and the bottom of the structure is set to be fixed. Initially, the smooth ice front is tangent to the structure surface and it moves along the structure horizontally at a constant speed of 0.15 m/s. The action process is ended after reaching the horizontal displacement to 9 m. The initial velocity of 0.15 m/s is set on the ice body element to avoid extra stress due to inertia in the flat ice. The ice buoyancy force induced by sea water is calculated based on the volume of ice unit submerged in sea water^37^.

$${F}_{B}=\left\{\begin{array}{*{20}l}0& z\ge 0\\ \frac{\rho g{V}_{e}z}{{l}_{e}}& -{l}_{e}\le z<{l}_{e}\\ \rho g{V}_{e}& z\le {l}_{e}\end{array}\right.$$

where ${F}_{B}$ is the ice buoyancy force, $\rho$ is the density of seawater, ${V}_{e}$ is the volume of ice unit, $z$ is the ordinate of integral point of the unit and ${l}_{e}$ is the physical size of ice unit. ${f}_{D}$ represents resistance of ice in water which is assumed to be related to the speed of ice movement^27^, as follows:

$${f}_{D}=\frac{1}{2}\rho {v}^{2}{S}_{c}{C}_{D}$$

where $v$ is the speed of ice in seawater, ${S}_{c}$ is the section area of the element and ${C}_{D}$ is the damping coefficient, ${C}_{D}=1.05$.

### Numerical results and validation

**Figure 4:**
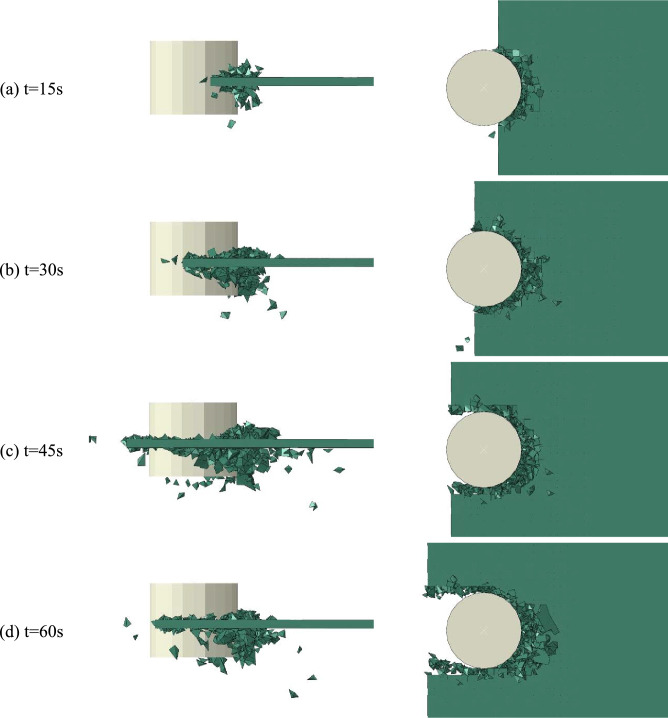
Partial magnification snapshots of interaction process.

As can be seen from Fig. 4, due to the small contact area between the flat ice and the structure at the initial contact stage, the flat ice produces small ice fragments under the extrusion action of the structure. The local extrusion and breakage of sea ice are reflected by plastic deformation of the ice body unit. Figure 4 also clearly shows the plastic deformation caused by ice body unit during collision and extrusion processes. Continuous invasion of structure in the ice and increase of contact area leads to a small amount of bending fracture as well as generation of large cracks with a certain length in the smooth ice. The ice-breaking channel profile in the figure is composed of circumferential cracks damaged by fracture, while the radial cracks produce broken ice pieces in various sizes and shapes. A few large broken ice pieces turn over and slide along the structure surface to the side of the model or under the water. These pieces finally return to the water surface under the combined action of buoyancy and gravity forces. Large accumulation of crushed ice, i.e., ice rubble can be clearly observed in front of the structure. The amount of ice rubble increases by continuous invasion of the structure. The broken ice produced in this process also interacts with the unbroken flat ice and affects the contact between the structure and the flat ice. The ice-breaking profile is formed by random extension of circumferential cracks on the ice breaking path, leading to an asymmetrical and irregular shape.

**Figure 5:**
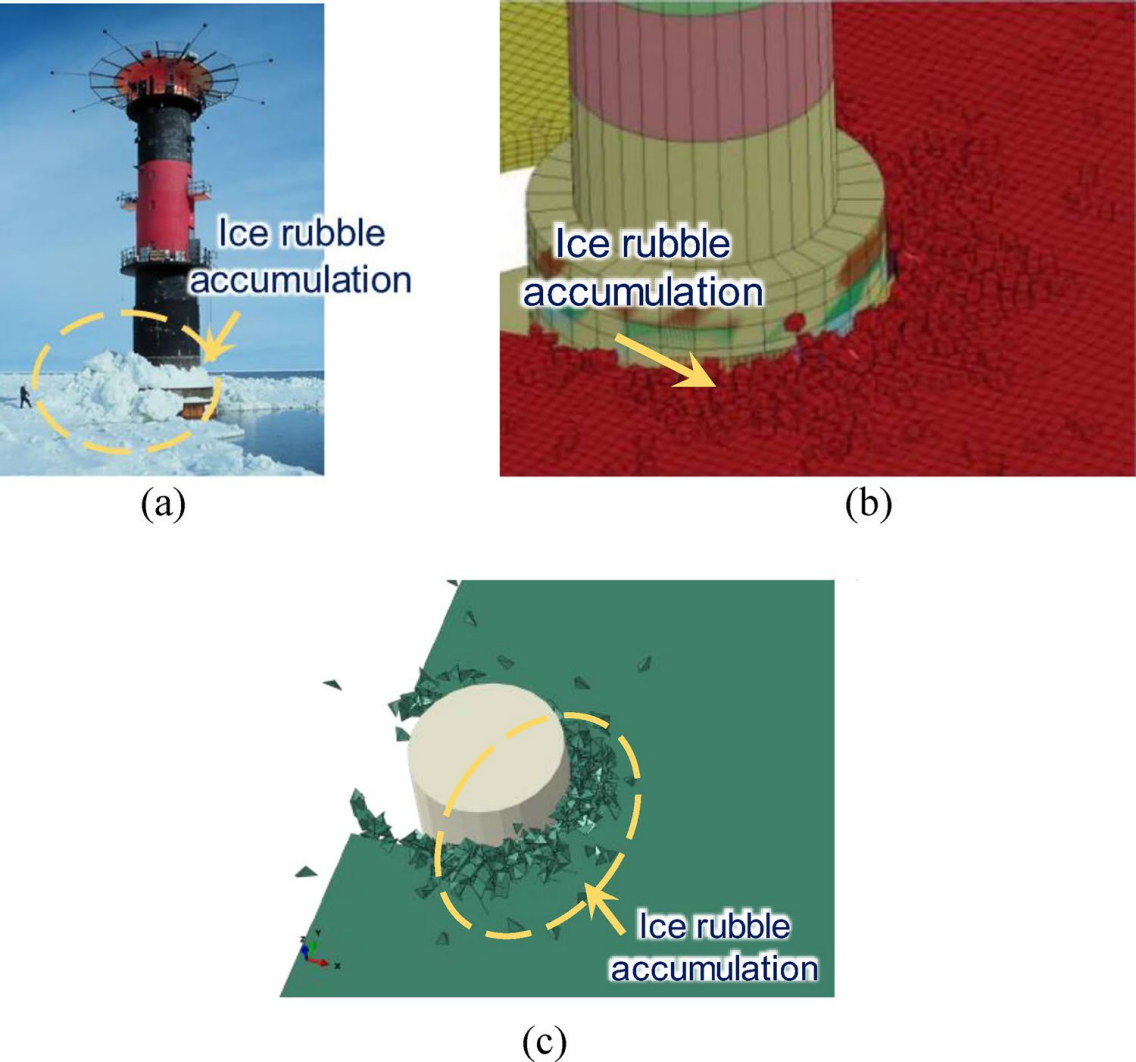
Ice failure at Norströmsgrund lighthouse: (**a**) Physical measurements^18^; (**b**) Numerical result by Hilding^33^; (**c**) This study.

Figure 5a,b compare the measured scene and simulation results of lighthouse structure and sea ice interaction with the results of Nord^18^ and Hilding^33^. The simulation results (Fig. 5c) are in good agreement with those reported by Hilding^33^. When the sea ice interacts with the structure at a lower speed, it mainly compresses and breaks along numerous micro-cracks. This forms a large number of broken ice pieces accumulating around the structure.

**Figure 6:**
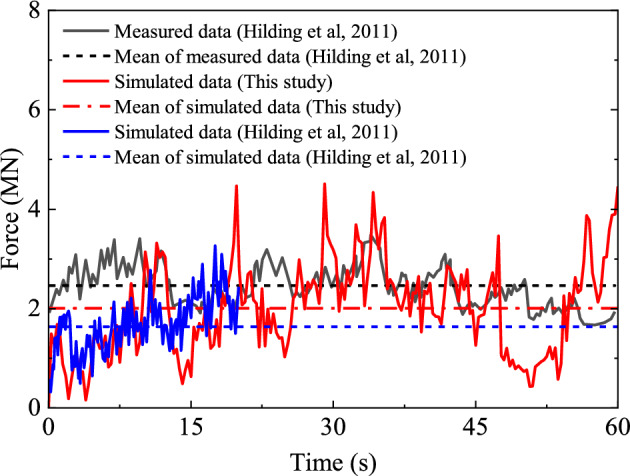
The simulated time histories of horizontal forces compared with the measurements.

The ice load time-history curve on the structure is extracted, as shown in Fig. 6. It can be seen from this figure that the load curve obtained by numerical simulation is in good agreement with the results of Hilding et al.^33^. Advancing the structure in the ice leads to steady squeeze of both the structure and the ice and growth of the load. At the same time, the ice is deformed under compression. By reaching the deformation to the critical value, the ice bending fails and a steep drop results in the load. This action is repeated by advancing the ice and results in the time-history curve of load which reveals oscillating trend of the result. Figure 6 also compares the average ice loads obtained from numerical simulation with those obtained in the literature. The average ice loads obtained from numerical simulations of the present study deviate by about 18.3% and 18.9% from the observations and the literature simulation results, respectively. Accordingly, very close results are obtained by numerical simulation for the average ice loads in comparison with the experimental results. However, it can also be seen from Fig. 6 that the peak value and fluctuation amplitude of the ice load time-history curve obtained by numerical simulation are slightly larger than the monitoring results. This may be due to different fracture lengths of sea ice in the numerical simulation and the actual problem. The collision process between marine structures and sea ice is random to some extent. Although the selected grid scale in numerical simulations is close to the radial fracture length of smooth ice, the simulated fracture length of sea ice cannot be exactly the same as the actual situation. The simulated fracture length may be slightly larger than the actual one, and therefore larger load peak value and load fluctuation amplitude are expected from the numerical simulation results.

## Effect of ice velocity and structure roughness

**Figure 7:**
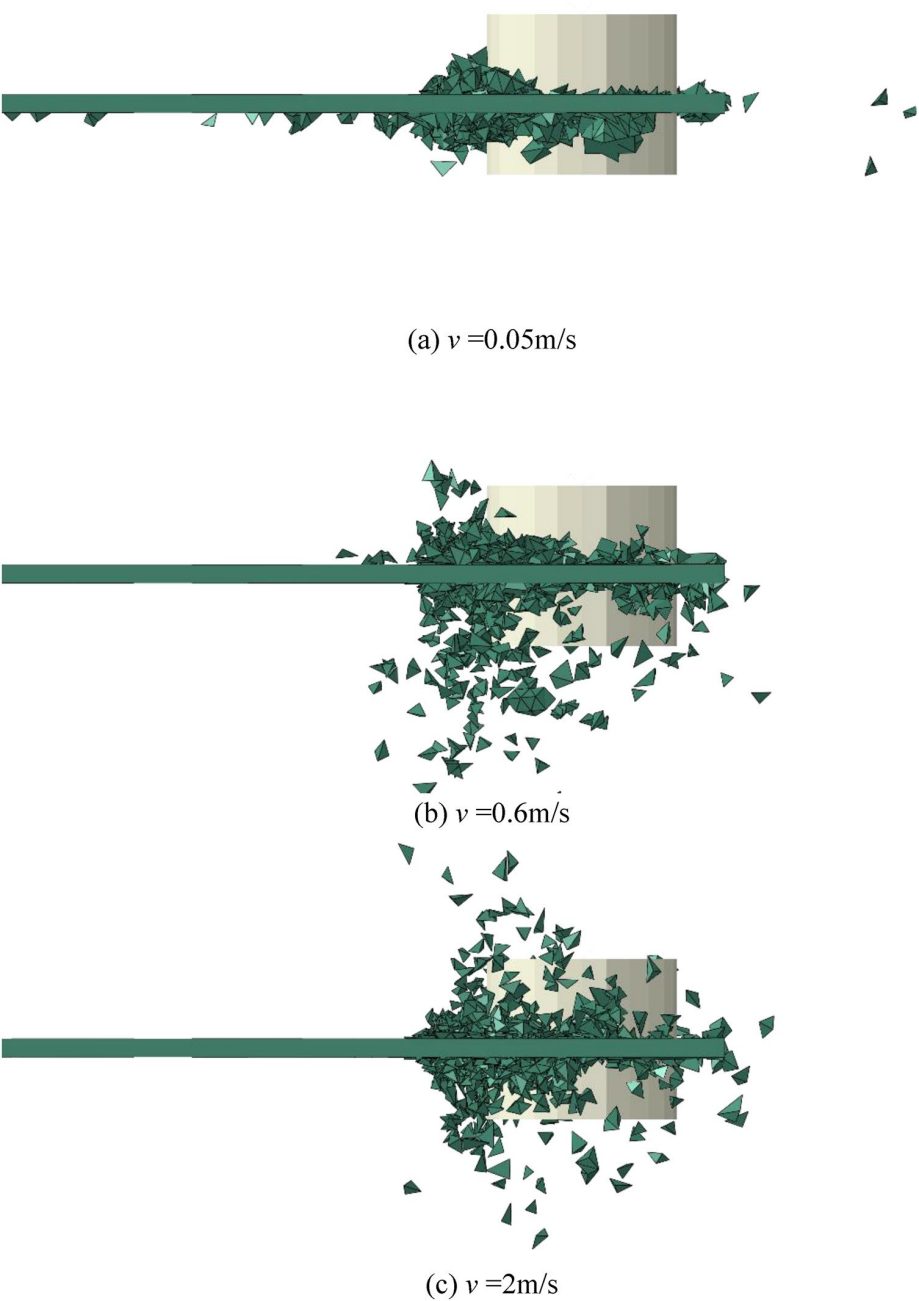
Partial magnification snapshots of ice failure under different velocity.

Sea ice movement speed is affected by wind speed, ocean current, and even observation means and conditions, etc. For example, sea ice movement speed in the Bohai Sea of China mostly ranges from 0.1 to 0.6 m/s, while sea ice drift speed in the Beaufort Sea of Canada ranges from 0.01 to 0.7 m/s, which is characterized by large fluctuations. Simulation is carried out based on different sea ice velocities in the range of 0.05–2 m/s. It should be noted that, for 0.05 m/s the results are only representative for very rigid structures as more flexible structures could experience ice-induced vibrations (intermittent crushing, frequency lock-in) for which the response of the structure matters and for which additional validation of the ice model would be needed^38^. The effect of sea ice velocity on ice fragmentation state as well as the impact of ice load on structures is examined. Figure 7 shows the sea ice fragmentation state after the ice-structure interaction for sea ice velocities of 0.05 m/s, 0.6 m/s and 2 m/s. It can be seen that during the ice-structure interaction at a lower speed, sea ice is mainly crushed and destroyed, and a large number of finely broken ice pieces are formed and accumulated at the structure sea level. With a further increase of the sea ice velocity to 0.6 m/s or even 2 m/s, the effect of sea ice on the structure is “impact” in which the sea ice is broken and separated obviously, or it can even be the “splash” in which a large number of isolated broken ice and floating ice pieces are formed. This result is consistent with observations of sea ice in the Beaufort Sea area as reported in Timco’s research^39^.

**Figure 8:**
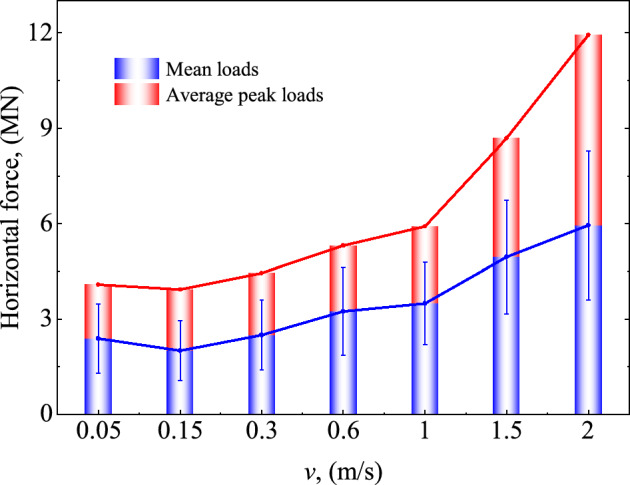
Ice load for different ice velocity.

The impact of horizontal ice load on the structure under different ice velocities is also examined, as illustrated in Fig. 8. The greater the sea ice velocity, the greater the ice load amplitude and average value on the structure. When the sea ice velocity *v* increases by 12 times (from 0.05 to 0.6 m/s) in the simulation, the average ice load and average peak load on the structure increase by 1.5 and 1.25 times, respectively. Growth of the sea ice velocity by 3.3 times (from 0.6 to 2 m/s) increases the average ice load and average peak load on the structure by 1.5 and 2.5 times, respectively. It is evident that the velocity impact on the ice load is more considerable for higher sea ice velocities. Combined with the results obtained in Fig. 7, the sea ice failure for velocities lower than 0.6 m/s is mainly caused by compression failure, and the sea ice velocity slightly affects the ice load. In contrast, sea ice velocity has a great influence on the ice load of the structure when the ice velocity is large (above 0.6 m/s). It should be pointed out that the sea ice speed in the actual condition will not exceed 1 m/s, and the response parameters here are mainly selected for a more intuitive comparison.

**Figure 9:**
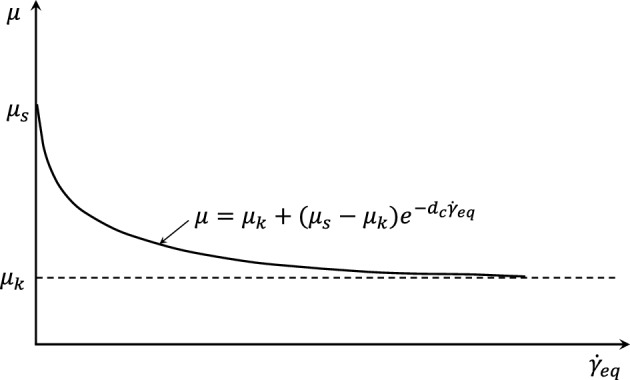
Exponential decay friction model.

**Figure 10:**
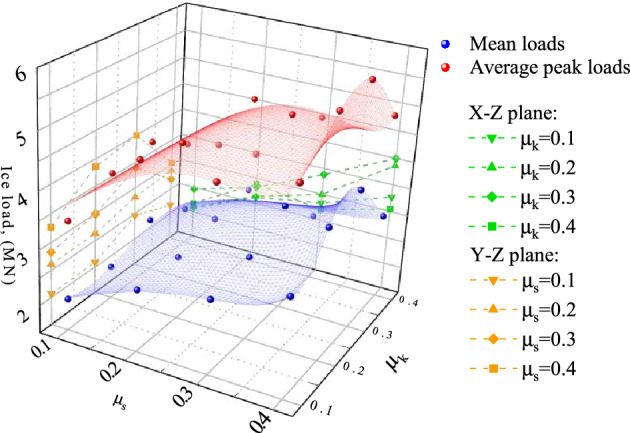
Force distribution with different friction factor.

Friction effect in the dynamic process is usually expressed by dynamic friction coefficient ${\mu }_{k}$ and static friction coefficient ${\mu }_{s}$. The exponentially attenuating friction model (Fig. 9) is adopted. The dynamic and static friction coefficients are selected within the range of 0.1–0.4. Totally, 16 working condition models are used to extract the average ice load and average peak load on the structure. Computational results are shown in Fig. 10.

Figure 10 indicates that the friction coefficient significantly affects the ice load on the structure. The ice load increases linearly with the increase of ${\mu }_{s}$. In contrast, the dynamic friction coefficient has a nonlinear effect on the structure ice load. The negative load increases by up to 86% for different values of ${\mu }_{k}$. When ${\mu }_{s}$ is constant, the structure ice load first exhibits an ascending trend and then it decreases with the increase of ${\mu }_{k}$. Under the selected working conditions, the structure ice load reaches its peak value when ${\mu}_{\text{k}}=\text{0.3}$. This difference may be related to friction model selection. In practical engineering problems, it is difficult to distinguish the dynamic or static friction coefficients of materials. However, computational results indicate that a smoother structure surface would be beneficial due to reduction of the ice load on the structure.

## Analysis of simulation results for different interfaces

**Figure 11:**
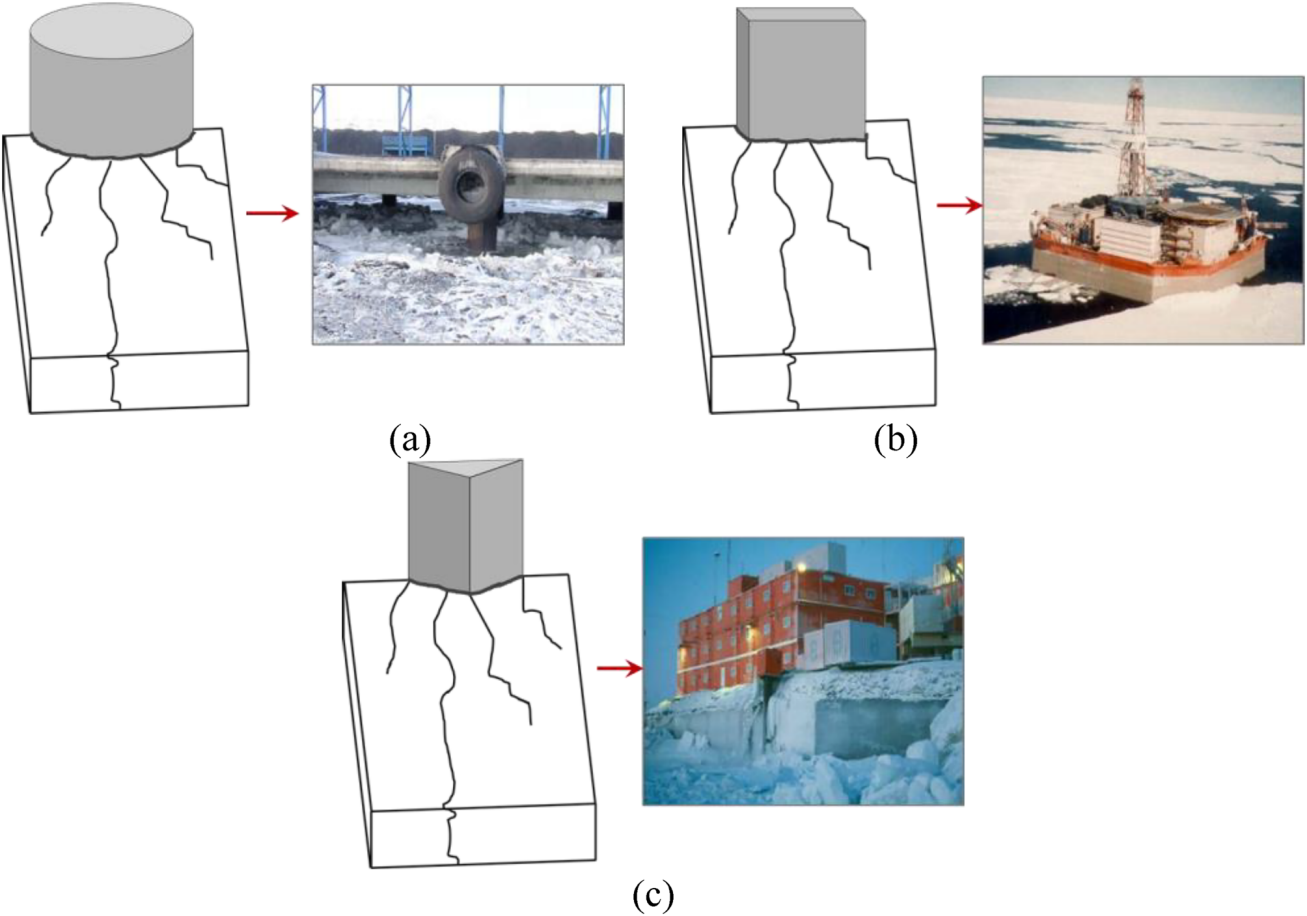
Study cases with different interface: (**a**) CS^40^; (**b**) PS^39^; (**c**) CI^39^.

**Figure 12:**
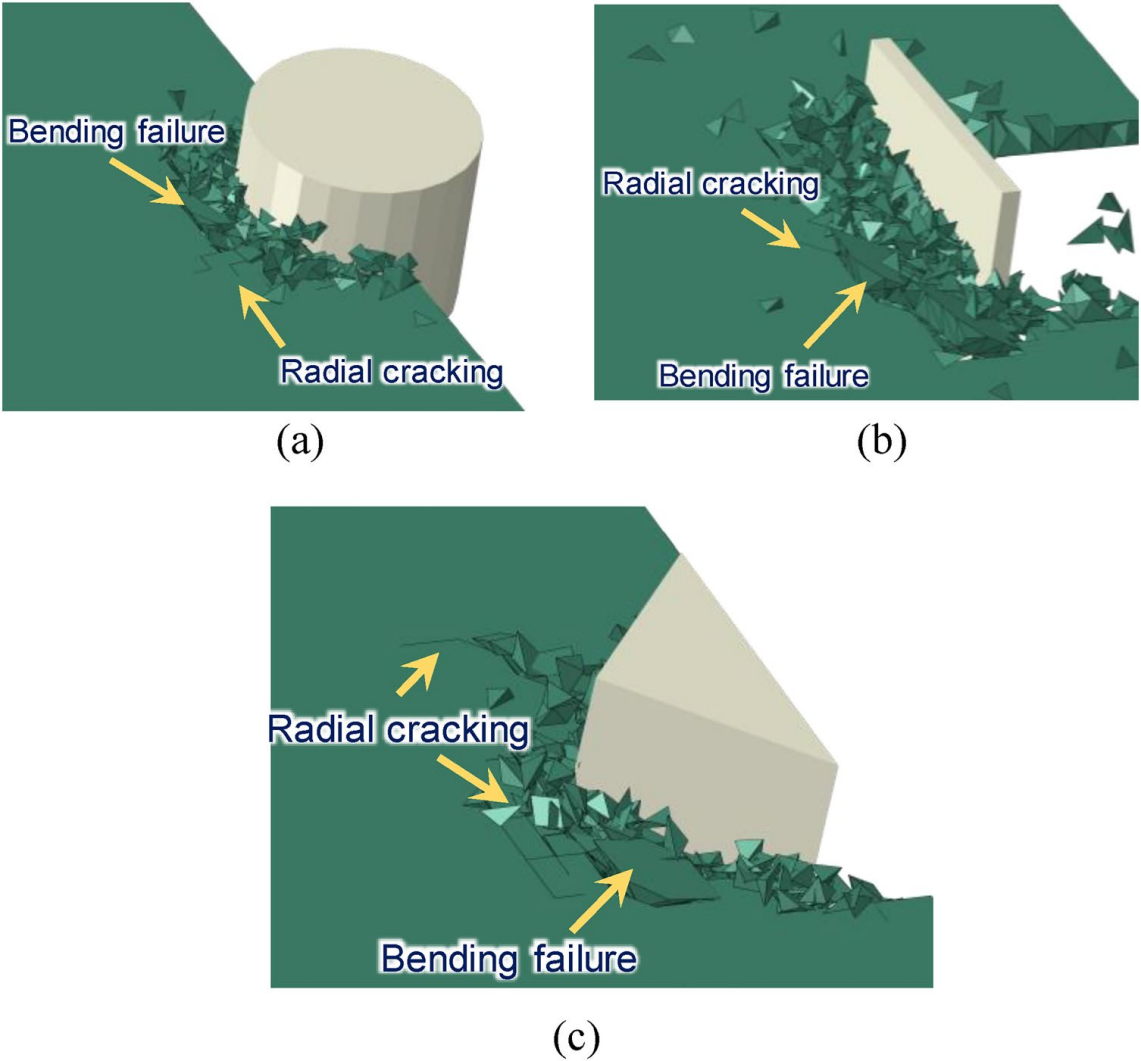
Partial magnification snapshot of failure ice: (**a**) CS; (**b**) PS; (**c**) CI.

Various structures have different geometric forms due to their functions or safety requirements. Therefore, the ice-structure interaction is also different due to diversity of structure shapes facing the ice surface. The common structural forms in polar engineering applications can be simplified into three types: cylindrical surface (CS), plate surface (PS) and corner interface (CI), as depicted in Fig. 11^39^^,^^40^. Interaction between sea ice and these three structural forms is compared in Fig. 12. In the case of cylindrical surface, it can be seen that the ice breakage is mainly caused by extrusion, and it results in a more uniform distribution due to the smoother contact surface between the CS and the ice. When the ice interacts with the PS structure, the ice exhibits a combination of extrusion and bending failures. In this case, crush failure is dominant in the middle of the interface, and bending failure occurs at the structure edge. This is mainly due to uneven contact between local ice and the structure, abrupt changes of internal stress, buoyancy and other unbalanced factors at the edge of the structure, which lead to the bending failure of sea ice. The ice load on the CI case is similar to that of the PS case. The sea ice exhibits a mixed fracture form consisting of crush and bending failures. The bending failure mainly occurs at the side of structure. Similar phenomenon (local crushing and global bending failures) was also observed in model tests^38,41^. And Gesa Ziemer^42,43^ observed intermittent crushing in model tests with different cross-section. which is considered to be superimposed by bending of the ice sheet, and the duration of intermittent crushing is longer in thicker ice.

In order to further explore the influence of different interfaces on ice load, parametric studies including the interface width, the inclination angle of interface and the angle of corner interface are conducted.

### Effect of the interface width

**Figure 13:**
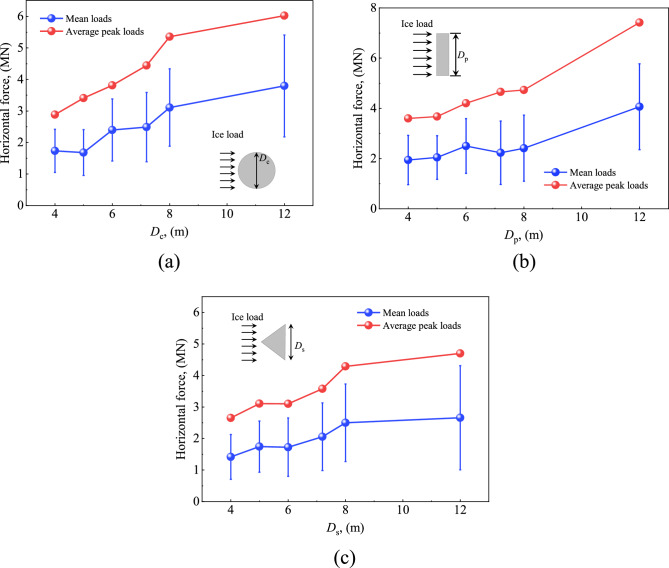
Ice load for different width of interface: (**a**) CS; (**b**) PS; (**c**) CI.

Figure 13 illustrates the ice load curves of the three structural forms at different projected widths of the interface (D). It can be seen that impact of the interface width on the ice load is basically the same for the three structural forms. A larger interface width leads to higher ice load on the structure with approximately linear growth trend. Standard deviation of the ice load also gradually increases. With the increase of D from 4 to 12 m, the ice load for CS, PS and CI cases increases by 105%, 101% and 70%, respectively. The main reason for this difference in the CI case may be incomplete contact of the ice with the structure at the beginning of the calculation. Accordingly, the load on the structure is small during this process, which results in a small average load in the whole process. Since the angle of CI configuration is constant in the present section, the structure height in the projection direction rises with the increase of the interface width. The longer the time for incomplete ice-structure contact, the greater the impact on the average load in the whole process.

### Effect of the inclination angle of interface

**Figure 14:**
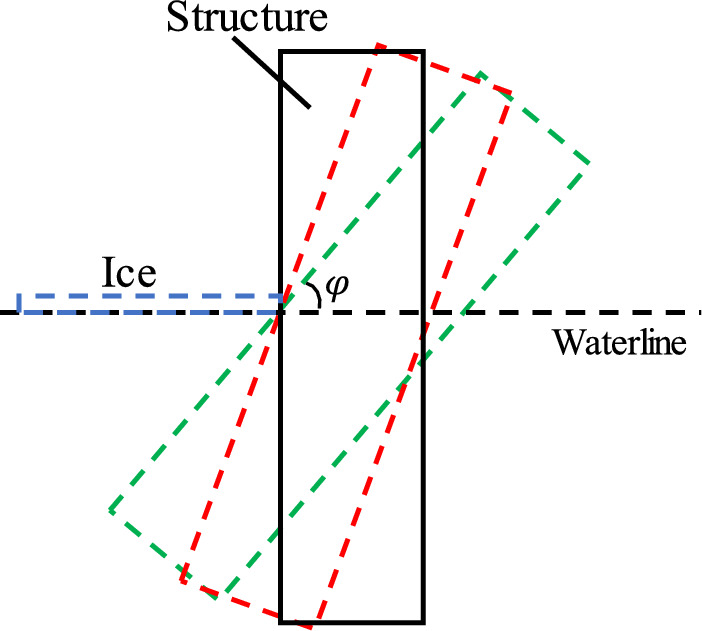
Illustration of structures with different inclined angles.

**Figure 15:**
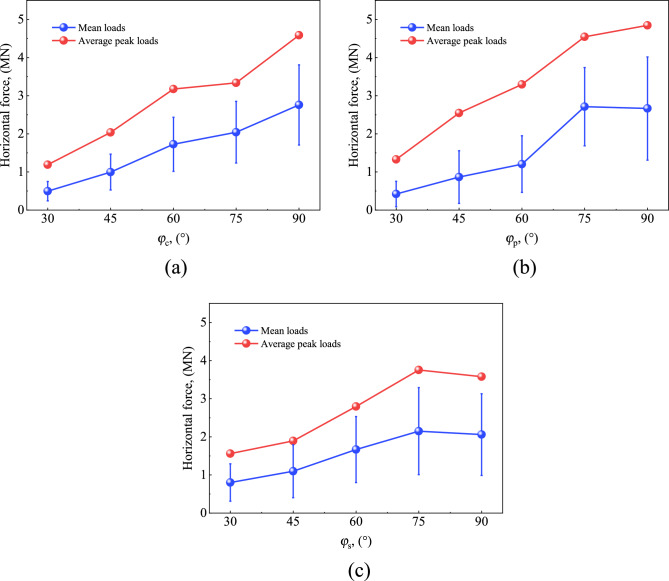
Ice load for different inclined angle of interface: (**a**) CS; (**b**) PS; (**c**) CI.

The definition of structural inclination φ is presented in Fig. 14. Five values are selected for simulation (φ = 30°, 45°, 60°, 75° and 90°). The structure height and the projected width at the surface line remained unchanged. Figure 15 shows a variation of the mean and standard deviation of ice load with φ based on numerical calculations. It can be seen that the mean value and standard deviation of ice load increase approximately linearly when φ increases from 30° to 75°. This is because the inclined plane of the structure becomes steeper with the increase of the dip Angle, and the broken ice pieces generated by the collision hardly slip away from the interface. As a result, more broken ice pieces are accumulated in front of the structure, leading to an increase of the ice load. This is consistent with the reported results of the ice accumulation load of inclined structures^27^.

### Effect of the interface corner angle

**Figure 16:**
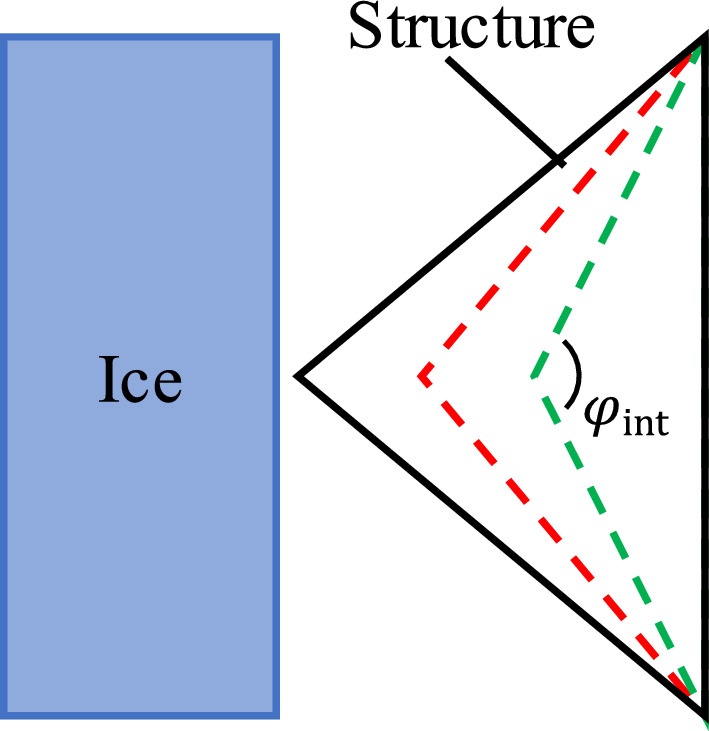
Illustration of structures with different sharp corners.

The definition of the interface corner angle ${ \varphi  }_{\text{int}}$ for the CI configuration is depicted in Fig. 16. Different cases with ${ \varphi  }_{\text{int}}=$ 45°, 60°, 75°, 90°, 120°, 150° and 180° are selected for calculations (${ \varphi  }_{\text{int}}={180^\circ}$ represents the plane facing the ice surface). The projected width of the structure remains unchanged.

**Figure 17:**
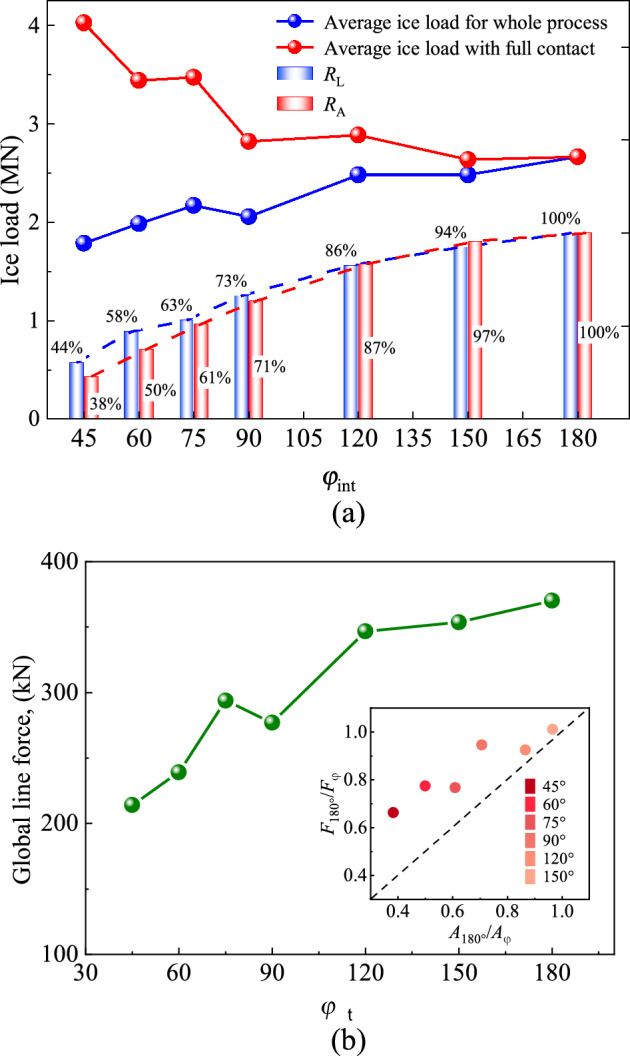
Influence of the angle of the sharp interface on ice load: (**a**) global load; (**b**) line load.

Similar to the above cases, when the projected width is constant, the larger value of ${ \varphi  }_{\text{int}}$ leads to smaller height of the structure section. Considering the influence of incomplete ice-structure contact on the ice load in the initial period of ice-structure interaction, the average ice loads in the whole interaction process and after reaching a complete ice-structure contact are evaluated, as shown in Fig. 17a. The results show that the ice load on the structure is related to the ice-structure interaction area, such that the larger the contact area, the greater the ice load on the structure. Furthermore, the smaller the interface corner angle, the larger the cross-section height of the structure. Therefore, the ice load on the structure decreases with the increase of ${ \varphi  }_{\text{int}}$ when the sea ice is in full contact with the structure. At the same time, the smaller ${\mathrm{\varphi }}_{int}$ leads to longer incomplete contact process of sea ice structure and smaller average ice load for the whole interaction process. Figure 17b presents variation of average line load on the structure with ${ \varphi  }_{\text{int}}$ under different working conditions. The average line load is defined as follows^39^:

$$ {\text{Line}}\,{\text{load }} = \frac{{{\text{Global}}\,{\text{ice}}\,{\text{load}}\,{\text{on}}\,{\text{structure}}}}{{{\text{Length}}\,{\text{of}}\,{\text{structure}}\,{\text{loaded}}\,{\text{by}}\,{\text{the}}\,{\text{ice}}}} $$

It can be seen that the average line load of ice increases linearly with the increase of ${ \varphi  }_{\text{int}}$. This is mainly because the side angle of the structure is relatively small when ${ \varphi  }_{\text{int}}$ is large, and it is more difficult for sea ice to slip away from the interface. As a result, more broken ice pieces are accumulated in front of the structure, leading to an increase of ice load.

## Load distribution along the ice interface

**Figure 18:**
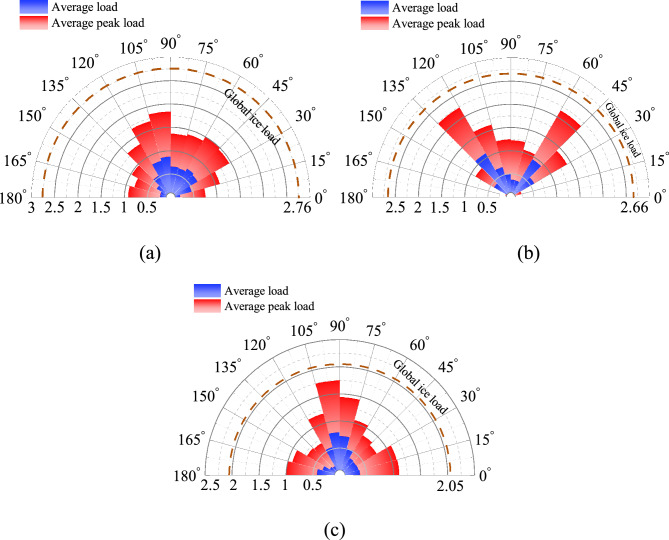
Ice load contribution on the interface (kN): (**a**) CS; (**b**) PS; (**c**) CI.

Figure 18 shows the rose diagram of ice load distribution in three structural forms. It can be found that the ice loads on the three structures are similar, and the global ice loads of the CI case are relatively small. Because of the different structural forms, the distribution of ice load on the structure is diverse. Ice load on the CS structure is mainly distributed in the middle of the contact area, while it is concentrated on both sides of the structure in the case PS shape. The load distribution of the CI case is similar to that of the CS case. The maximum load appears in the middle of the interface, and the load on both sides of the structure is larger than that of the CS case.

**Figure 19:**
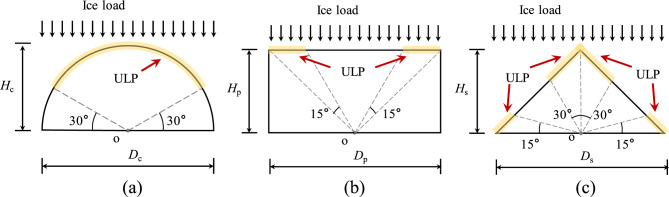
Diagram of ice load for different interface: (**a**) CS; (**b**) PS; (**c**) CI.

The ice load distribution of the three structural geometries can be simplified to the form shown in Fig. 19. The regions marked in yellow are the unfavorable load position (ULP). The ratios of the ULP length to the interface length for CS case, PS case and CI case are 66.7%, 42.3% and 57.4%, respectively. Meanwhile, the ratios of the ice loads on ULP to the total ice load on the structures for these three cases are 72.1%, 61.5% and 78.4%, respectively. It is obviously that ULP region bears most of the ice load, and the CS structure has higher material utilization while the corner position of PS structure and CI structure would be posed on higher risk. In engineering applications, the load distribution characteristics of different structural forms can be targeted to strengthen the design.

## Summary and conclusions

In this paper, the interaction between a moving level ice sheet and fixed structures with different interfaces was simulated by CEM. 3D numerical models were developed in ABAQUS and validated by in-situ testing data and results of a previous numerical model. The buoyancy and damping models were implemented through user-defined subroutines. Then, a series of simulations concerning ice velocity, structure roughness, interface width, inclination angle of interface and corner angle of interface were conducted. The ice load and its distribution on the structure were analyzed. The main conclusions can be expressed as follows:

1. Sea ice velocity has a significant influence on ice failure mode and ice load. With the increase of the sea ice velocity to 0.6 m/s or even 2 m/s, the effect of sea ice on the structure is “impact” resulting in obvious breakage and separation of the sea ice, or it can even exhibit “splash” in which a large number of isolated broken ice and floating ice pieces are formed.
2. The static and dynamic friction coefficients show different effects on ice load. The ice load increases linearly with the growth of ${\mu }_{s}$, while ${\mu }_{k}$ has a nonlinear effect on the structure ice load. A smoother structure surface would be beneficial in the reduction of the ice load on the structure.
3. Ice load magnitude is related to the ice-structure contact area. Both larger width and larger inclination angle would increase the global ice load on the structure. Examination of cases with different interface corner angles indicates dependence of the ice load on the distance between the structure and the ice sheet.
4. Different ice load distribution patterns were observed in various interfaces. In the CS case, the ice load on the structure is mainly distributed in the middle of the contact area which shows higher material utilization. The corner position of PS structure and CI structure is subjected to large ice load with less material, thus would be posed on higher risk.

### Supplementary Information

Supplementary Video 1.

## Data Availability

The datasets used and/or analysed during the current study available from the corresponding author on reasonable request.

## References

[1] Timco GW, Weeks WF (2010). A review of the engineering properties of sea ice. Cold Reg. Sci. Technol..

[2] Boroojerdi MT, Bailey E, Taylor R (2020). Experimental study of the effect of submersion time on the strength development of freeze bonds. Cold Reg. Sci. Technol..

[3] Qu Y, Yue Q, Bi X, Kärnä T (2006). A random ice force model for narrow conical structures. Cold Reg. Sci. Technol..

[4] Yue Q, Qu Y, Bi X, Kärnä T (2007). Ice force spectrum on narrow conical structures. Cold Reg. Sci. Technol..

[5] Dalane O (2014). Influence of pitch motion on level ice actions. Cold Reg. Sci. Technol..

[6] Lu W, Lubbad R, Løset S (2014). Simulating ice-sloping structure interactions with the cohesive element method. J. Offshore Mech. Arct. Eng..

[7] Long X, Liu S, Ji S (2020). Discrete element modelling of relationship between ice breaking length and ice load on conical structure. Ocean Eng..

[8] Long X, Liu S, Ji S (2020). Breaking characteristics of ice cover and dynamic ice load on upward–downward conical structure based on DEM simulations. Comput. Particle Mech..

[9] Timco GW, Irani MB (1992). Model tests of dynamic ice loading on the Chinese JZ-20-2 jacket platform. Can. J. Civil. Eng..

[10] Timco, G. W., Wright, B. & Johnston, M., et al. First-year ice ridge loads on the Molikpaq. In *Proceedings of the 4th International Conference on Development of the Russian Arctic Offshore*. St. Petersburg, Russia (1999).

[11] Timco GW, Johnston M (2004). Ice loads on the caisson structures in the Canadian Beaufort Sea. Cold Reg. Sci. Technol..

[12] Timco GW (2011). Isolated ice floe impacts. Cold Reg. Sci. Technol..

[13] Timco GW, Sudom D (2013). Revisiting the Sanderson pressure-area curve: Defining parameters that influence ice pressure. Cold Reg. Sci. Technol..

[14] Johnston, M. & Timco, G. W. Ice Loads on the SSDC during Its Beaufort Sea Deployments. In *Proceedings of the International Conference on Port and Ocean Engineering Under Arctic Conditions*, Trondheim, Norway (2003).

[15] Wright BD, Timco GW (2001). First-year ridge interaction with the Molikpaq in the Beaufort Sea. Cold Reg. Sci. Technol..

[16] Frederking, R., Timco, G. W. & Wright, B. Ice pressure distributions from first-year sea ice features interacting with the Molikpaq in the Beaufort Sea. In Proceedings of the 9th International Offshore and Polar Engineering Conference, Ottawa, Canada (1999).

[17] Palmer AC, Dempsey JP, Masterson DM (2009). A revised ice pressure-area curve and a fracture mechanics explanation. Cold Reg. Sci. Technol..

[18] Nord TS, Øiseth O, Lourens E (2016). Ice force identification on the Norströmsgrund lighthouse. Comput. Struct..

[19] Nord TS, Samardžija I, Hendrikse H (2018). Ice-induced vibrations of the Norströmsgrund lighthouse. Cold Reg. Sci. Technol..

[20] Shi, C. Investigation on mechanism of marine structure-sea ice collision using finite element method. Ph. D thesis. Shanghai Jiao Tong University (2017).

[21] Gürtner, A. Experimental and numerical investigations of ice-structure interaction. Ph. D thesis. Norwegian University of Science and Technology (2009).

[22] Xiao, J. Damage and fracture of brittle viscoelastic solids with application to ice load models. Ph. D thesis. Memorial University of Newfoundland (1997).

[23] Barenblatt GI (1962). The mathematical theory of equilibrium cracks in brittle fracture. Adv. Appl. Mech..

[24] Mulmule SV, Dempsey JP (1997). Stress-separation curves for saline ice using fictitious crack model. J. Eng. Mech..

[25] Patil A, Sand B, Cwirzen A (2021). Numerical prediction of ice rubble field loads on the Norströmsgrund lighthouse using cohesive element formulation. Ocean Eng..

[26] Kuutti J, Kolari K, Marjavaara P (2013). Simulation of ice crushing experiments with cohesive surface methodology. Cold Reg. Sci. Technol..

[27] Wang F, Zou Z, Zhou L (2018). A simulation study on the interaction between sloping marine structure and level ice based on cohesive element model. Cold Reg. Sci. Technol..

[28] Wang Y, Zou Z, Wang F (2019). A simulation study on the ice fracture behaviors in ice-lighthouse interaction considering initial defects in ice sheet. Ocean Eng..

[29] Määttänen M, Marjavaara P, Saarinen S (2011). Ice crushing tests with variable structural flexibility. Cold Reg. Sci. Technol..

[30] Feng, D., Pang, S. D. & Zhang, J. Parameter sensitivity in numerical modelling of ice-structure interaction with cohesive element method. In *Proc. Int. Conf. On Ocean, Offshore and Arctic Engineering*, Busan, South Korea (2016).

[31] ISO19906. Petroleum and Natural Gas Industries - Arctic Offshore Structures (2010).

[32] Kulyakhtin S, Høyland KV (2015). Ice rubble frictional resistance by critical state theories. Cold Reg. Sci. Technol..

[33] Hilding, D., Forsberg, J. & Gürtner, A. Simulation of ice action loads on offshore structures. In 8th European LS-DYNA Users Conference, Strasbourg, France (2011).

[34] Hilding, D., Forsberg, J. & Gürtner, A. Simulation of loads from drifting ice sheets on offshore structures. Proceedings of the 12th International LS-DYNA Users Conference, Detroit, Michigan (2012).

[35] Ervik Å, Nord TS, Høyland KV, Samardzija I, Li H (2019). Ice-ridge interactions with the norströmsgrund lighthouse: Global forces and interaction modes. Cold Reg. Sci. Technol..

[36] Albrektsen, A. Prediction of the response from ice forces to a lighthouse structure, Master thesis, Department of Structural Engineering, NTNU (2009).

[37] Valanto, P., Jones, S.J., Enkvist, E., Izumiyama, K. The resistance of ships in level ice. In Discussion. Author’s closure. Transactions—Society of Naval Architects and Marine Engineers. 109, 53–83 (2001).

[38] Hendrikse H, Ziemer G, Owen CC (2018). Experimental validation of a model for prediction of dynamic ice-structure interaction. Cold Reg. Sci. Technol..

[39] Timco GW, Johnston M (2003). Ice loads on the Molikpaq in the Canadian Beaufort Sea. Cold Reg. Sci. Technol..

[40] Løset S, Aleksey M (2009). Field studies and numerical simulations of ice bustles on vertical piles. Cold Reg. Sci. Technol..

[41] Hammer TC, Hendrikse H (2023). Experimental study into the effect of wind-ice misalignment on the development of ice-induced vibrations of offshore wind turbines. Eng. Struct..

[42] Ziemer, G., Evers, K. U. & Voosen, C. Influence of structural compliance and slope angle on ice loads and dynamic response of conical structures. In *International Conference on Offshore Mechanics and Arctic Engineering*. American Society of Mechanical Engineers (2015).

[43] Ziemer, G. Ice-induced vibrations of vertical structures. Ph. D thesis. Technische Universität Hamburg (2021).

