# Author Correction: Numerical study of ice loads on different interfaces based on cohesive element formulation

**DOI:** 10.1038/s41598-023-42776-w

**Published:** 2023-09-15

**Authors:** Wenqiang Xing, Shengyi Cong, Xianzhang Ling, Xinyu Li, Zhihe Cheng, Liang Tang

**Affiliations:** 1 School of Civil Engineering, Harbin Institute of Technology, Harbin, 150090 Heilongjiang China; 2 State Key Laboratory of Frozen Soil Engineering, Cold and Arid Regions Environmental and Engineering Research Institute, Chinese Academy of Sciences, Lanzhou, 730000 Gansu China

Correction to: *Scientific Reports* 10.1038/s41598-023-41618-z, published online 02 September 2023

The original version of this Article omitted an affiliation for Shengyi Cong. The correct affiliations are listed below.

‘1. School of Civil Engineering, Harbin Institute of Technology, Harbin, Heilongjiang 150090, China.’

‘2. State Key Laboratory of Frozen Soil Engineering, Cold and Arid Regions Environmental and Engineering Research Institute, Chinese Academy of Sciences, Lanzhou, 730000, Gansu, China.’

The original Article has been corrected.

